# Potential impacts of climate-related decline of seafood harvest on nutritional status of coastal First Nations in British Columbia, Canada

**DOI:** 10.1371/journal.pone.0211473

**Published:** 2019-02-27

**Authors:** Lesya Marushka, Tiff-Annie Kenny, Malek Batal, William W. L. Cheung, Karen Fediuk, Christopher D. Golden, Anne K. Salomon, Tonio Sadik, Lauren V. Weatherdon, Hing Man Chan

**Affiliations:** 1 Biology Department, University of Ottawa, Ottawa, Ontario, Canada; 2 Nutrition Department, Faculty of Medicine, Université de Montréal, Pavillon Liliane de Stewart, Montreal, Québec, Canada; 3 Changing Ocean Research Unit, Institute for the Oceans and Fisheries, University of British Columbia, Vancouver, British Columbia, Canada; 4 Nippon Foundation-UBC Nereus Program, Institute for the Oceans and Fisheries, University of British Columbia, Vancouver, British Columbia, Canada; 5 Dietitian and Nutrition Researcher, Victoria, British Columbia, Canada; 6 Department of Environmental Health, Harvard TH Chan School of Public Health, Boston, Massachusetts, United States of America; 7 Harvard University Center for the Environment, Cambridge, Massachusetts, United States of America; 8 School of Resource & Environmental Management, Simon Fraser University, Burnaby, British Columbia, Canada; 9 Assembly of First Nations, Ottawa, Ontario, Canada; 10 UN Environment World Conservation Monitoring Centre, Cambridge, United Kingdom; Swedish University of Agricultural Sciences and Swedish Institute for the Marine Environment, University of Gothenburg, SWEDEN

## Abstract

**Background:**

Traditional food systems are under pressure from various stressors, including climate change which is projected to negatively alter the abundance of marine species harvested by coastal First Nations (FNs) in British Columbia (BC).

**Objective:**

To model the potential impacts of the climate-related declines in seafood production on the nutritional status of coastal BC FNs. In addition, we projected potential changes in nutrient intakes, under different scenarios of substitution where traditional seafood is replaced with alternative non-traditional foods.

**Methods:**

The study design is a mixed-method approach that combines two datasets: projected scenarios of climate-related change on seafood catch potential for coastal BC FNs and data derived from the cross-sectional First Nations Food, Nutrition, and Environment Study. The consumption of seafood was estimated using a food frequency questionnaire among 356 FNs. The contribution of seafood consumption to protein, eicosapentaenoic acid (EPA) and docosahexaenoic acid (DHA), vitamins (A, B12, D, niacin), and minerals (zinc, selenium and iron) requirements was assessed using Dietary Reference Intakes (DRIs).

**Results:**

Traditional seafood consumption provided daily recommendations of EPA+DHA (74–184%) and vitamin B12 (84–152%) and substantial levels of niacin (28–55%), selenium (29–55%), vitamin D (15–30%) and protein (14–30%). Projected climate change was estimated to reduce the intakes of essential nutrients by 21% and 31% under ‘strong mitigation’ (Representative Concentration Pathway, RCP2.6) and ‘business-as-usual’ (RCP8.5) climate change scenarios, respectively, by the year 2050 relative to 2000. The hypothetical substitution of seafood with selected alternative non-traditional foods does not provide adequate amounts of nutrients.

**Conclusion:**

Traditionally-harvested seafood remains fundamental to the contemporary diet and health of coastal BC FNs. Potential dietary shifts aggravated by climate-related declines in seafood consumption may have significant nutritional and health implications for BC FN. Strategies to improve access to seafood harvest potential in coastal communities are needed to ensure nutritional health and overall well-being and to promote food security and food sovereignty in coastal FNs.

## Introduction

Marine environments support the livelihoods and diets of billions of people worldwide [1]. Fish is a rich source of essential micronutrients [2], with over 4.5 billion people deriving at least 15% of their average intake of animal protein from fish [3]. Globally, changes in ocean temperature and acidity are projected to impact marine species distribution, affecting fisheries yields, catch composition, and revenue [4–7]. Climate-related declines in fish catch are also projected to mediate knock-on impacts on human health, through the decline of access to critical micronutrients in the diets of seafood-dependent peoples [8]. Eleven-percent of the current global population is estimated to become vulnerable to poor nutrition from climate-related fisheries declines [8]. This raises significant equity concerns as the majority of countries that are highly seafood-dependent are also low-income, food-deficient, countries [9]. Moreover, at the sub-national level, these risks may be experienced more strongly in certain populations, such as among coastal Indigenous peoples, where fish consumption greatly exceeds national averages [10], and among whom, significant disparities in socioeconomic and health status exist [11,12].

This paper is focused on the traditional food systems of coastal First Nations in British Columbia (BC). First Nations (FNs) are among the original inhabitants of the land and sea that is now considered Canada. Within Canada, there are three distinct Indigenous populations: First Nation, Inuit and Métis. The FN population includes individuals who may or may not be members of a specific FN and hold a registered or treaty Indian status. In the most recent National Census (2016), 1,673,785 or 4.9% of the total population in Canada identified as Indigenous (i.e. First Nations, Inuit and Métis), while 977,230 or 2.8% of the total population identified as First Nation and 744,855 (76.2% of total FNs) indicated that they are registered or Treaty Indian status [13]. Registered Indians are persons who are registered under the Indian Act of Canada. Across Canada, there are 634 unique First Nations/Indian Bands, with one-third (n = 203) located in the province of British Columbia (BC) [14,15]. Most FNs have tracts of land held by the Crown that fall under the reserve system and are classified as “Indian Reserve” or IR. Among the 744,855 FNs with Registered Treaty Status, 42% (312,839) reside on IR [13]: there are 135,835 Status Indians in BC, with approximately 51,000 living on-IR.

FNs in Canada have sustained themselves for millennia through diverse resource management and food production technologies including hunting, foraging, trapping and intensive food production (clam gardens, estuarine root beds, berry patches, crab-apple orchards, species domestication including sunflower, corn, beans and squash). [16–22]. These local foods have been collectively called “traditional foods” [16], and are highly valued for their contribution to well-being [17,20,23].

FNs have been undergoing significant disparities in socioeconomic and health status resulting in a transition in diet and lifestyle during the last century [24–26]. This nutrition transition, characterized by moving away from healthy traditional foods towards less healthy store-bought foods, has detrimental health effects. FNs in Canada have a lower life expectancy, higher rates of mortality, and a greater burden of chronic diseases [26,27], relative to the non-Indigenous population of Canada. Cardiovascular disease among BC FNs is almost double (7.9% vs. 4.8%) the rate among the general BC population [24,28], and many more BC FNs are overweight (36.6%), obese (36%) or have diabetes (9.9%) [29].

Furthermore, BC FNs are facing high rates of food insecurity (i.e. the inability to afford nutritionally adequate and safe foods). Overall, 41% of BC FN’s households on reserves are food insecure (33% are defined as moderately food insecure while 8% are considered to be severely food insecure (skipping or cutting the size of their meals)) [30]. This is in contrast with the provincial average, where less than ten percent (8.4%) of households in BC (excluding FN’s households on reserves) experience food insecurity [31]. Food insecurity among Indigenous Peoples in Canada including BC FN has been associated with a compromised diet quality, low intake of essential nutrients, poorer health, and low mental status [32–34]. Restricted access to traditional foods and the subsequent erosion of cultural harvesting traditions today can also be attributed to centralized colonial fisheries regulations [35]. With respect to fisheries, regulations pertaining to saltwater species, and salmon fishing in freshwater fall under the federal “Fisheries Act”, while freshwater fisheries are provincially regulated. For fisheries falling under federal jurisdiction, a fisheries strategy has been in place since 1992 and allocates specific amounts of fish, namely salmon, that can be harvested by FNs for Food, Social and Ceremonial (FSC) purposes [36,37]. FNs have repeatedly argued that FSC allocations are inadequate to meet their needs and subsequently profoundly impact both diet (for example, current allocations may be equivalent to 5 salmon per capita) and other factors that influence well-being, including economic opportunities [38,39].

In the Nuxalk FN in BC, the consumption of spring salmon decreased from 38 Kg/family/year to 13 Kg/family/year, and sockeye decreased from 27 Kg/family/year to 5 Kg/family/year between 1981 and 2009 [40]. This decline in fish and seafood has stemmed from many social, economic and environmental factors [30,31]. European colonization is considered to be as a fundamental underlying driver of the nutrition and lifestyle transition among FNs [25]. The loss of traditional territories, as well as forced attendance of residential schools, led to the erosion of FNs’ culture, loss of language, reduced access to traditional food sources and diminished traditional knowledge transition [11,30,32,33]. Socio-economic barriers, such as poverty, unemployment, high cost and limited variety and availability of healthy market foods, as well as high cost of fishing equipment, contribute to the nutrition and food insecurity in Indigenous communities [23,34,35]. Under these conditions, many FNs turn to cheap low-nutrient market foods [16,30].

Despite significant lifestyle and environmental changes, traditional food systems remain fundamental to the culture, livelihood, economy, and health of contemporary FNs [16]. Even when consumed in small amounts, traditional foods provide significant sources of energy, protein, essential vitamins, minerals, and long-chain omega-3 fatty acids (n-3 FAs) as well as other health benefits, including improvements in mental health, and maintenance of healthy connections to many cultural dimensions (identity, cultural practices, sense of place)[41–43]. Coastal FNs, in particular, rely on a wide variety of locally harvested marine foods (fish, shellfish, and seaweeds) and salmon, a cultural keystone species [44] for their diet [19,30].

Traditional food systems are under pressure from various stressors, including climate change, which is projected to negatively impact the abundance and catch potential of many marine species harvested by BC FNs [45]. Fisheries declines may result in the decrease in the availability of seafood which is an important source of nutrients for BC FNs, given that their seafood consumption is much higher than the rest of the population in general [10]. Although such decrease in wild-caught seafood availability may be compensated by purchasing food from other sources, previous studies on Indigenous people’s diet suggest that such shifts are often towards increased consumption of processed (and other) energy-dense foods that are high in fat, refined sugar, and sodium [41,46–48]. A recent report that assessed Canadian fish stocks suggested that more than 50% of the assessed fish stocks in the Pacific coast are considered to be fully- or over- exploited. Under such a scenario, it is likely that fisheries decline as a result of climate change would further exacerbate the risk of food insecurity and the “nutrition transition” (i.e., the substitution of nutrient-rich traditional foods with store-bought foods of high energy-density but limited nutritional value) among FNs in BC. However, the burden of climate change on seafood consumption and FN nutritional status has not been quantified. Previous research on the impact of climate change on traditional food systems and adaptation planning has mainly been conducted in the Arctic regions, focusing on Inuit and northern FNs [49–52]. Published research on climate change effects on FNs living in southern and coastal regions of Canada is limited. However, one First Nation, the Gitga’at Nation, located on BC’s north coast has been actively engaged in climate-related research and its impacts on seasonal harvesting and indicators [53], incorporating local values and knowledge into creating robust climate change adaptation strategies [54].

**Fig 1:**
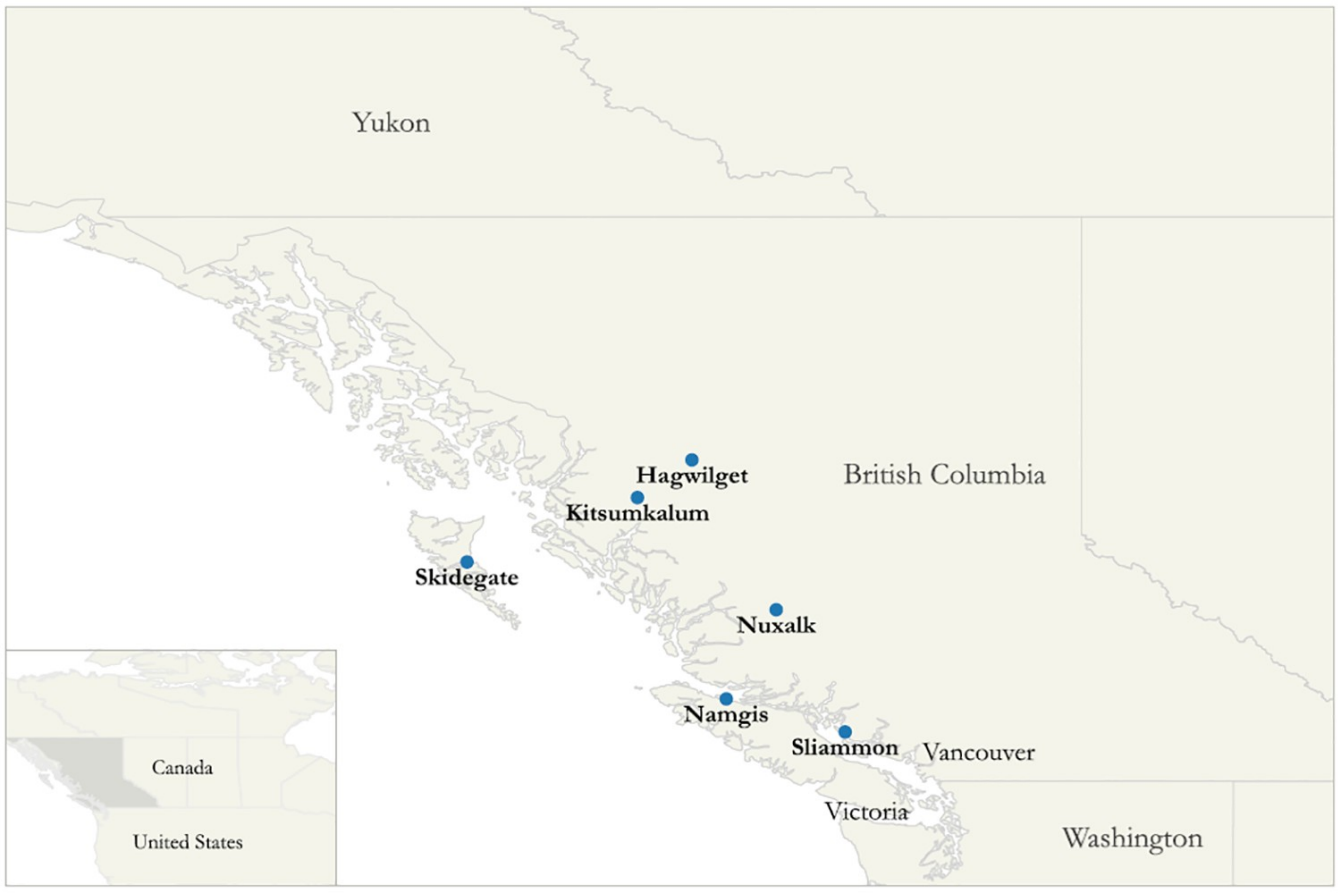
Map of participating First Nations communities in British Columbia.

The objective of this study was to estimate the potential impacts of the climate-related declines in seafood abundance on the nutritional quality of the diets of FN adults in BC living on IR in coastal communities in Canada (Fig 1), based on a climate model projecting changes in potential fish catch and known FN dietary data. In addition, we projected potential changes in nutrient intakes, after assuming the hypothetical substitution of traditional seafood with alternative non-traditional foods.

## Methodology

The study’s design, adapted from [8], is a mixed-methods approach which integrates methods and data from two traditionally distinct research fields: marine ecology and human nutrition. The approach explores the relationship between changes in fisheries catch potential and human nutrition and aims to assess the impacts of climate-related declines in seafood harvest on human diets and nutrient intakes. To this end, two datasets were used: i) projected scenarios for coastal catch potential under climate change in FNs fisheries [45]; and ii) dietary data from the First Nations Food, Nutrition and Environment Study (FNFNES) in BC [30]. Weatherdon et al. [45] quantitatively projected impacts of climate-related change on the relative abundance, geographic range distribution and richness of 98 seafood species of commercial and cultural importance to FNs in coastal BC. The changes were modelled under two emission scenarios: the low emission ‘strong mitigation’ (Representative Concentration Pathway (RCP) 2.6) and high emission ‘business-as-usual’ (RCP 8.5) by 2050 relative to 2000 [55]. The FNFNES is a cross-sectional survey which was designed to assess traditional food consumption, total diets, food insecurity, and food-related exposure to environmental contaminants in FNs living on IR, south of the 60^th^ parallel across Canada. The FNFNES (www.fnfnes.ca) collected data from approximately 100 FNs south of the 60^th^ parallel in 10 provinces, including the six coastal BC FNs, over a 10-year period (2008–2017) and is representative of all Canadian FN regions [56].

### Projected effects of climate change on seafood abundance

We used published projections of the effects of climate change on the relative abundance of 98 marine fish and invertebrate species of commercial and cultural importance to FNs in coastal BC [45]. These projections were based on outputs from the dynamic bioclimate envelope model (DBEM), which is a mechanistic species distribution model that simulate changes in annual average abundance (relative to baseline time period) on a 0.5 ° latitude x 0.5° longitude grid of the world ocean [45,57]. Briefly, the DBEM includes four different components: (1) temperature and oxygen-dependent size, growth and mortality [58,59], (2) estimation of population carrying capacity in each grid cell based on environmental conditions and species’ habitat preferences, which can increase or decrease according to changes in environmental conditions and the species’ habitat preferences, (3) modelled ocean conditions dependent spatial population dynamics with adult movement and (4) larval dispersal modelled using an advection-diffusion-reaction model (see [57] for detailed description of the model). Changes in seafood abundance were estimated under RCP 2.6 and RCP 8.5, representing “strong mitigation” and “business-as-usual” scenario. Changes in ocean conditions under climate change scenarios were projected from the Geophysical Fluid Dynamics Laboratory Earth System Model 2M (GFDLESM 2M) [60]. Projected changes in ocean conditions were regridded on a 0.5° latitude x 0.5° longitude grid of the global oceans on which the DBEM operates (see [57] for details). Total annual average abundance in the Pacific Coast of Canada (within Canada’s Exclusive Economic Zone) were calculated for each species. The results for the 2050s period (average between 2041 to 2060) relative to the 2000s (average between 1991 to 2010) were then used in subsequent analysis. Despite the uncertainties associated with the coarse-scale (relative to the BC FN fisheries) projections, these represent one of the best available species-specific quantitative projections in the literature that cover a wide range of species that are important to BC FNs. We also discussed the implications of the uncertainties associated with the use of these projections for the conclusions of this study.

In total, 98 species comprising marine and diadromous fish, shellfish, and invertebrates harvested by First Nations for food, social, and cultural purposes were selected. The list of species was identified from peer-reviewed literature, government and non-governmental organizations’ reports, treaty agreements, and First Nations’ reports. Marine mammals, seaweeds and birds were not included in the study [45].

### Dietary data from the FNFNES

A total of 21 FNs in British Columbia were surveyed between the Fall of 2008 and 2009. FNs were randomly selected using a combined ecozone/cultural area framework and residency information from on-reserve populations. An ecozone is a large geographical region identified by the distribution patterns of plants, animals, geographical characteristics and climate (ecozone.ca). The cultural area is a concept used to identify geographic areas within which Indigenous communities share a greater number of traits/cultural affinities than those outside the area [61]. This study uses the data obtained from the six FNs communities in the Pacific Maritime ecozone, Subarctic/Northwest coast cultural area, including Kitsumkalum, Hagwilget, Skidegate, Nuxalk, Namgis, and Tla’amin (formerly Sliammon) (Fig 1). The Pacific Maritime ecozone comprises BC marine islands, the land along the Pacific Coast, and the border with Alaska. A total of 549 randomly selected households in the 6 randomly selected communities in the Pacific Maritime ecozone were invited to participate. The participation rate was 67%. In total, 369 individuals (one participant randomly selected from each household) who self-identify as FN adults living on-reserve were recruited to participate in this study. Pregnant and breastfeeding women (n = 13) were excluded from our analyses due to their different nutritional requirements and low sample size. The final sample included 356 individuals. All the data were weighted to obtain representative estimates of the total population by minimizing biases arising from differences between participating and non-participating persons using the Bootstrap method [30]. Ethical approval was granted by the Ethical Review Boards at Health Canada, the University of Northern British Columbia, the University of Ottawa and the Université de Montréal.

Data on age, sex, weight, height, physical activity level, smoking behaviour, educational attainment, household size, employment status, self-perceived health status, source of income, body mass index, and traditional food gathering activity were collected. Food security information was obtained using the income-related Household Food Security Survey Module (HFSSM) [62] adapted from the United States Department of Agriculture (USDA) Food Security Survey Module [63]. The HFSSM was further adapted for FNs [64]. Dietary information was collected by a 24-hour diet recall and a Traditional Food Frequency Questionnaire (FFQ) spanning the entire year and collecting information on the frequency of consumption of all traditional foods available for consumption. In total, the FFQ combined 208 traditional food items including 65 seafood species. Seafood species included fish (n = 41), shellfish (n = 16), seaweed (n = 4), and marine mammals (n = 4) reported in the survey.

### Estimation of nutrient intakes and nutrient requirements

Each food item reported to be consumed in the dietary intake assessments were matched to the Canadian Nutrient File, a national food composition database [65] taking into account the preparation method (i.e. baked or broiled, boiled or raw). Nutrients that are rich in seafood were selected to be included this study: protein, eicosapentaenoic acid (EPA) and docosahexaenoic acid (DHA), vitamins (A, D, B12, and niacin), and minerals (zinc and selenium, and iron). See Supporting Information S1 Table for nutrient content of top 20 most consumed seafood species.

We used the Dietary Reference Intake (DRI), in particular, the Recommended Dietary Allowance (RDA), to assess the contribution of seafood consumption to nutrient requirements [66]. The DRIs are a comprehensive set of nutrient values for healthy populations used for assessing and planning diets. The RDA is the average daily dietary nutrient intake level that is sufficient to meet the nutrient requirements of nearly all (97.5%) healthy individuals in a particular life stage and gender group [67]. The RDAs were available for all nutrients of interest except EPA and DHA. According to the Dietitians of Canada and the American Heart Association, consumption of 500mg/d EPA+DHA is recommended for the general adult population without cardio-heart diseases (CHD) which is considered to be sufficient to obtain protective effects for primary prevention of cardiovascular diseases [68,69]. Thus, the contribution of EPA+DHA intake to the recommended intake (RI) was estimated. Nutrient requirements are the same for those aged 19 to 30 years and those aged 31 to 50 years; for this reason, these age groups were combined. Age groups containing those between 51 and 70 (inclusive) and those over 70 years of age were also combined due to the small sample size among the elderly.

### Statistical analyses

Data management and statistical analyses were performed using STATA statistical software, 14.2 (StataCorp, College Station, Texas, USA). Descriptive statistics included the calculation of means with standard deviations (SD) for continuous variables (age, BMI, years of education, household size and seafood consumption) and proportions for categorical variables (sex, smoking, dieting, physical activity, health status, unemployment, food insecurity, income sources and traditional activity). Student t-tests were performed to assess whether differences in seafood consumption (total and by species) between sex and age groups were statistically significant. The top 20 most consumed seafood species were determined by ranking the mean intake (mean (95% CI)) of food items. The top 20 species represented almost 90% of total seafood intake, by weight. Ranks were performed for the total population, and for men and women, respectively given the significant sex differences in seafood consumption. Mean daily intakes of selected nutrients from the top 20 seafood species were estimated. The percentage contributions of total seafood consumption to the nutrient requirements (DRAs and RI) were calculated according to sex and age groups [67,70]. The mean proportion method was used to estimate percentage contribution of each food species to the DRIs [71]. All estimates were weighted in order to obtain representative data at the regional level.

### Combining projected climate effects and food use data

Using data on the projected scenarios of seafood harvest decline [45], we modelled the potential impact on nutritional adequacy among coastal FNs in BC. Declines in relative species abundance were calculated under the lower (RCP 2.6) and upper (RCP 8.5) scenarios of climate change using 20-year average abundances for 2050 (2041–2060) relative to 2000, within FN’s domestic fishing areas (DFA). The FN’s DFAs are the geographical areas used for harvesting fish and aquatic plants for food, social and ceremonial purposes. The approximate size of each FN’s DFA was derived from the Statement of Intent (SOI) boundaries registered with the BC Treaty Commission as of October 2004, which were converted to 0.5° latitudinal by 0.5° longitudinal grid-cells of the ocean to correspond with the grid system of DBEM. While these boundaries do not signify the full extent of territory previously used by First Nations, particularly with respect to the sharing of resources between communities, they serve to illustrate the approximate areas requested by First Nations for food, social, ceremonial, and commercial fishing purposes [45].

The average change of all seafood species was calculated as a sum of weighted contributions of all species to total seafood intake (by weight). The obtained average projection of all seafood species was assumed to represent the changes for species with no data. Changes in nutrient intakes and their contributions to the nutrient recommendations were estimated by subtracting the expected percent change in seafood consumption under RCP 2.6 and 8.5 from the baseline nutrient intakes. In addition, changes in nutrient intakes were projected after substitution (gram-to-gram) of seafood with alternative foods. For this initial stage of exploring potential impacts, three hypothetical scenarios were developed using simplistic replacement of lost seafood with market foods reported to be among the top 10 for protein, market fish and energy in the 24-hr recall results that are: 1) an alternative land protein source (chicken), 2) an alternative marine protein source (canned tuna), and 3) an energy source (bread) [30]. These foods are not suggested alternatives but simply serve to illustrate possible nutrient intake under hypothetical substitution scenarios.

## Results

A total of 356 individuals (140 men and 216 women) were included in this study. Demographic and lifestyle characteristics of the study population are summarized in Table 1. The mean age (± standard deviation, SD) of participants was 48.4 (±14.0) years old. The mean BMI was 30.9 (±7.1) kg/m^2^ (which is classified as obese (BMI ≥30kg/m^2^)) according to the World Health Organization [72] with 25.0% of FNs overweight, and 59.8% obese. In the general Canadian population, 17.9% of adults aged 18 years and older were obese while 36.1% were overweight [73] Smoking cigarettes was reported by 41% among both men and women. The prevalence of unemployment was 33% while 35% of respondents reported food insecurity. For comparison, in 2009, 20.1% of Canadians 12 years and older were daily or occasional smokers [74] whereas 8.7% were unemployed [75]. Approximately 71% of individuals participated in traditional food gathering, with 34% reporting fishing, and 23% collecting beach food. Overall, coastal BC FNs consumed 56.3 (±72.5) g/d (grams/person/day, g/d) of seafood (unweighted estimates) (Table 1).

**Table 1:** Descriptive characteristics of coastal First Nations in British Columbia.

	mean/n	SD/%
n	356	100
Age, year (mean, SD)	48.4	14
Female (n, %)	216	61
Smoking (n, %)	145	41
Dieting (n, %)	44	12
BMI (kg/m2) (mean, SD)	30.9	7.1
Physical inactivity (n, %)	66	18
Health status (n, %)		
excellent/very good	99	28
good	141	39
fair/poor	116	33
Years of education (mean, SD)	11.1	2.7
Household size (no. of people) (mean, SD)	3.1	1.9
Unemployment (n, %)	116	33
Food insecurity (n, %)	126	35
Income sources (n, %)		
wages	193	54
social assistance	99	27
workers compensation	15	4
pension	49	14
Traditional activity (n, %)		
any	251	71
fishing	121	34
collecting seafood	82	23
Seafood consumption (g/d) (mean, 95%CI)	56.3	48.7, 64.9
Seafood consumption (19-50y) (mean, 95%CI)	52.0	43.9,60.0
Seafood consumption (50+y) (mean, 95%CI)	62.1	48.1,76.1

SD, Standard Deviation; %, percent; n, number; unweighted estimates

Dieting (on the day before the interview) in order to lose weight

Traditional activity, traditional food gathering activity by participants

Food insecurity combines moderate and severe food insecurity

Pregnant and lactating women were excluded (n = 13)

The percentage of consumers (total, and by men and women) and mean intake of the top 20 species as well as total seafood consumption among coastal FNs in BC, are presented in Table 2. Salmon (all species combined) were the most commonly consumed seafood. Halibut and herring roe were the second and fourth most consumed species, followed by shellfish (prawn, clam, crab, and shrimp). The proportion of respondents consuming at least one out of the top 20 most consumed seafood species ranged between 21% and 86% and was comparable between men and women (p values >0.05). Mean intake (95% CI) for each of the top 20 seafood species, except herring roe, was higher among males than female. Overall, the top 20 species represented about 87% of men’s total seafood intake and 88% of women’s intake, by weight.

**Table 2:** Top 20 most consumed seafood species in coastal First Nations in British Columbia, ranked by from greatest to least mean intake (total)^a^.

	Total			Men			Women		
Seafood	%	mean	95% CI	%	mean	95% CI	%	mean	95% CI
Sockeye salmon	85	12.20	6.4, 18.0	85	18.0	6.5, 29.4	86	9.6	5.7, 13.6
Halibut	82	5.81	2.9, 8.7	82	6.4	2.5, 10.2	83	5.6	2.8, 8.3
Chinook salmon	57	3.95	1.3, 6.6	62	5.8	1.8, 11.9	54	3.1	1.5, 4.7
Herring roe	62	3.01	2.1, 3.0	52	2.0	1.3, 6.6	67	3.4	1.5, 2.4
Coho salmon	54	2.54	0.5, 5.5	55	3.9	0.9, 3.1	53	1.9	0.5, 6.4
Prawn	53	2.24	0.2, 4.4	55	2.9	1.1, 4.6	51	2.0	0.3, 4.3
Clam	67	2.20	1.0, 3.3	66	2.3	1.8, 2.8	68	2.1	0.6, 3.5
Salmon egg	41	2.11	0.1, 4.2	44	2.5	0.5, 4.6	38	1.9	0.2, 4.0
Chum salmon	42	2.10	0.5, 3.1	46	2.6	1.1, 4.7	38	1.9	0.4, 2.4
Crab	59	1.82	1.6, 1.9	59	2.9	1.0, 1.9	59	1.4	1.7, 2.1
Shrimp	46	1.80	1.7, 1.9	33	1.5	1.0, 1.9	52	2.0	1.7, 2.2
Eulachon grease	58	1.64	0.2, 3.1	45	1.5	0.3, 2.7	64	1.7	0.1, 3.3
Pink salmon	33	1.56	0.8, 2.3	36	2.8	0.1, 5.5	32	1.0	0.0, 2.0
Rockfish	40	1.54	1.3, 1.8	47	3.1	1.3, 4.8	37	0.9	0.6, 1.1
Ling cod	29	1.45	0.7, 2.2	42	1.2	0.8, 1.6	22	1.6	0.6, 2.4
Eulachon	53	1.34	0.4, 2.3	43	1.9	0.8, 2.9	58	1.1	0.2, 2.0
Black cod	28	1.03	0.1, 2.0	34	1.2	0.1, 2.2	25	1.0	0.0, 1.9
Pacific cod	28	1.01	0.4, 1.6	25	1.4	0.3, 2.5	29	0.8	0.4, 1.2
Basket cockle	50	0.93	0.6, 1.3	44	1.0	0.4, 1.5	53	0.9	0.5, 1.3
Trout, any	25	0.76	0.1, 1.4	21	1.7	0.6, 4.1	26	0.3	0.2, 0.5
Top 20 combined	98	52.5	37.9, 67.2	98	68.6	37.0, 100.2	99	45.4	35.0, 55.9
Total seafood	99	59.9	40.3, 79.4	98	78.7	38.2, 119.2	99	51.5	37.1, 65.9

^a^—Data from the First Nation Food, Nutrition and Environment Study in British Columbia (2008–09), FFQ questionnaire, Individuals aged 19 years+

%—percent consumers of a respective food; mean intake (g/person/day) of each food item based on the sample of 351 (out of 356) consumers of seafood;

Top 20 seafood species represented 87% (males), and 88% (females) of total seafood intake, by weight; weighted estimates

Overall, 99% of all participants reported eating seafood in the prior year. Total mean seafood consumption was 78.7 (95% CI: 38.2–119.2) g/day among men and 51.5 (95% CI: 37.1–65.9) g/day among women. Older participants (>50 years of age) reported significantly higher seafood consumption (80.2 (95% CI: 44.5–115.8)) compared to younger individuals (between 19 and 50 years of age) (41.6 (95% CI: 28.6–54.5)) (p<0.05) (weighted estimates).

Dietary consumption data for 57 kinds of seafood was collected by the FNFNES. Six food items in the consumption data were different parts (organs, fat and eggs) and 13 species were not consumed by FN participants in BC, leaving a total of 38 species. Projected abundance (biomass) data were available for 29 distinct species (Table 3). When DBEM projections were available for sub-species (i.e., humpback shrimp, side-striped shrimp, humpy shrimp or redstripe rockfish, canary rockfish, shortraker rockfish, quillback rockfish, widow rockfish, etc.), average group level change was calculated based on the arithmetic mean of the projections for its composite species assemblage. The DBEM projections were not available for 9 seafood species, including four seaweed species (laver, kelp, rockweed, sea lettuce), two marine mammals (sea lion, harbour seal), and three beach foods (china slipper (gumboot chiton), octopus, sea prune (black chiton). The seafood species without projections contributed only 6.5% to the total seafood consumption (seaweed contributed 5% whereas other species– 1.5%).

**Table 3:** Projected changes in relative abundance of seafood species under lower (RCP 2.6) and upper (RCP 8.5) scenario of climate change in coastal First Nations in British Columbia by 2050 relative to 2000*. All species are predicted to decrease with the exception of kelp greenling under RCP 8.5.

	Difference (%)	
Seafood	lower	upper
Shrimp	46.1	64.1
Herring	31.8	48.7
Chinook salmon	47.8	46.8
Pink salmon	40.3	44.1
Eulachon	26.4	37.6
Sockeye salmon	10.2	36.2
Pacific cod	12.6	35.0
Starry flounder/English sole	21.6	28.9
Dolly Varden trout	10.8	28.1
Sea urchin	13.6	25.9
Mussel	10.6	23.9
Prawn	12.4	18.1
Oyster	17.8	18.3
Cutthroat trout	9.4	16.6
Coho salmon	8.8	15.2
Sea cucumber	12.8	14.9
Abalone	10.5	13.9
Crab	12.8	9.7
Rockfish	7.9	9.2
Halibut	12.3	13.0
Chum salmon	9.6	12.1
Scallops	8.0	11.2
Basket cockle	12.6	11.1
Barnacle	11.7	10.8
Clams	9.3	4.9
Black cod	10.8	9.2
Lingcod	8.7	7.3
Kelp greenling	7.7	+2.2

* Lower and upper scenarios of climate change represent the low and high greenhouse gas emission scenarios based on evidence of latitudinal and regional trends. Declines in relative abundance were projected by 2050 (relative to 2000) for seafood species within British Columbia’s marine environment under both scenarios of climate change [45].

Projected effects on relative abundance of seafood species are summarized in Table 3. We ranked seafood species according to the percentage decrease in relative abundance by 2050 relative to 2000. Shrimp, herring, chinook and pink salmon were projected to experience the greatest relative impact of climate change under both RCPs (34% to 60% declines). Sockeye salmon, the major contributor to the total seafood consumption, was projected to decline by 10% to 36% by 2050 relative to 2000. Halibut, the second-most consumed seafood, was estimated to decline by about 12% to 13%. The projected decline in abundance of different shellfish species ranged from 8% to 23% under lower and upper scenarios. We estimated that the average climate-related decline of all seafood species by biomass consumed by FNs was 21% and 31% under RCP 2.6 and 8.5, respectively.

The current intake of selected nutrients for men and women are presented in Tables 4 and 5, respectively. Nutrients analyzed include protein, n-3 FAs (EPA+DHA), vitamins (A, B12, D, and niacin), and minerals (zinc, selenium, and iron) derived from the top 20 types of seafoods individually and, combined, as well as all for men and women separately. Overall, males had higher intakes of selected nutrients because they consumed more seafood than females; however, these difference in nutrient intakes were only statistically significant (p<0.05) for EPA+DHA. The contribution of the top 20 types of seafood to total seafood-related nutrient intake ranged from 50% to 92% (men), and 53 to 92% (women) for different nutrients (by weight).

**Table 4:** Mean daily intake of selected nutrients by First Nations men, derived from the top 20 types of seafoods^a^.

	Protein	EPA+DHA	vitamin D	vitamin A	vitamin B12	Niacin	Zinc	Selenium	Iron
Seafood	g	mg	μg	μg RAE*	μg	mg NE"	mg	μg	mg
Sockeye salmon	4.57	221.14	2.36	12.41	1.02	1.74	0.09	6.56	0.09
Halibut	1.43	15.24	0.30	1.52	0.08	0.50	0.03	3.52	0.01
Chinook salmon	1.50	101.51	0.75	8.69	0.17	0.87	0.03	2.73	0.05
Herring roe	0.45	47.19	0.24	1.63	0.16	0.04	0.02	0.81	0.01
Coho salmon	0.92	41.47	0.44	2.00	0.20	0.48	0.02	1.49	0.02
Prawn	0.50	5.02	0.00	1.77	0.02	0.05	0.04	1.08	0.01
Clam	0.59	6.61	0.00	3.98	0.45	0.19	0.06	1.49	0.07
Salmon eggs	0.69	60.98	0.00	0.00	0.00	0.14	0.02	0.00	0.02
Chum salmon	0.55	29.99	0.17	0.46	0.11	0.18	0.03	1.11	0.02
Crab	0.64	11.36	0.00	0.89	0.30	0.10	0.16	1.37	0.01
Shrimp	0.33	4.09	0.00	1.31	0.02	0.04	0.02	0.72	0.00
Eulachon grease	0.00	15.03	0.00	0.32	0.00	0.00	0.00	0.00	0.00
Pink salmon	0.64	29.88	0.40	0.55	0.14	0.32	0.03	1.10	0.02
Rockfish	0.68	10.58	0.14	0.15	0.05	0.24	0.01	2.34	0.01
Ling cod	0.28	3.21	0.00	0.21	0.05	0.08	0.01	0.57	0.01
Eulachon	0.28	58.61	0.00	0.17	0.00	0.08	0.00	0.00	0.02
Black cod	0.20	20.91	0.00	1.19	0.02	0.10	0.00	0.55	0.02
Pacific cod	0.27	2.27	0.01	0.03	0.03	0.07	0.01	0.40	0.00
Basket cockle	0.26	2.84	0.00	1.71	0.20	0.08	0.03	0.64	0.00
Trout, any	0.45	15.90	0.08	0.32	0.13	0.18	0.01	0.28	0.03
Top 20 combined	15.21	703.83	4.01	39.32	3.15	5.87	0.85	26.74	0.43
Total seafood	17.80	817.83	4.49	47.83	3.63	7.55	1.21	28.92	0.86
% top 20 to total seafood	85%	86%	89%	82%	87%	78%	70%	92%	50%

^a^—Data from the First Nation Food, Nutrition and Environment Study in British Columbia (2008–09), FFQ questionnaire, Individuals aged 19 years+

*—Retinol activity equivalents, RAE;

"—Niacin equivalents, weighted estimates

**Table 5:** Mean daily intake of selected nutrients by First Nations women, derived from the top 20 types of seafoods^a^.

	Protein	EPA+DHA	vitamin D	vitamin A	vitamin B12	Niacin	Zinc	Selenium	Iron
Seafood	g	mg	μg	μg RAE*	μg	mg NE"	mg	μg	mg
Sockeye salmon	2.45	118.46	1.26	6.65	0.55	0.93	0.05	3.52	0.05
Halibut	1.25	13.36	0.27	1.34	0.07	0.44	0.02	3.08	0.01
Chinook salmon	0.80	54.26	0.40	4.65	0.09	0.46	0.02	1.46	0.03
Herring roe	0.77	80.62	0.42	2.79	0.28	0.06	0.03	1.39	0.02
Coho salmon	0.45	20.52	0.22	0.99	0.10	0.24	0.01	0.74	0.01
Prawn	0.34	3.45	0.00	1.22	0.02	0.03	0.02	0.74	0.00
Clam	0.55	6.10	0.00	3.67	0.42	0.17	0.06	1.37	0.06
Salmon eggs	0.52	46.14	0.00	0.00	0.00	0.10	0.01	0.00	0.01
Chum salmon	0.41	22.30	0.13	0.34	0.08	0.13	0.02	0.82	0.01
Crab	0.30	5.33	0.00	0.42	0.14	0.05	0.07	0.64	0.01
Shrimp	0.44	5.47	0.00	1.76	0.03	0.05	0.03	0.97	0.01
Eulachon grease	0.00	17.03	0.00	0.36	0.00	0.00	0.00	0.00	0.00
Pink salmon	0.24	11.01	0.15	0.20	0.05	0.12	0.01	0.40	0.01
Rockfish	0.19	2.96	0.04	0.04	0.01	0.07	0.00	0.65	0.00
Ling cod	0.35	4.08	0.00	0.26	0.06	0.10	0.01	0.73	0.01
Eulachon	0.16	34.13	0.00	0.10	0.00	0.05	0.00	0.00	0.01
Black cod	0.17	17.87	0.00	1.02	0.01	0.08	0.00	0.47	0.02
Pacific cod	0.15	1.32	0.00	0.02	0.02	0.04	0.00	0.23	0.00
Basket cockle	0.23	2.57	0.00	1.55	0.18	0.07	0.02	0.58	0.00
Trout, any	0.09	3.25	0.02	0.07	0.03	0.04	0.00	0.06	0.01
Top 20 combined	9.87	470.21	2.17	27.43	2.01	3.89	0.51	16.60	0.28
Total seafood	12.17	511.35	2.43	31.69	2.25	4.93	0.71	17.99	0.53
% top 20 to total seafood	81%	92%	89%	87%	89%	79%	72%	92%	53%

^a^—Data from the First Nation Food, Nutrition and Environment Study in British Columbia (2008–09), FFQ questionnaire, Individuals aged 19 years+

*—Retinol activity equivalents, RAE;

"—Niacin equivalents, weighted estimates

**Fig 2:**
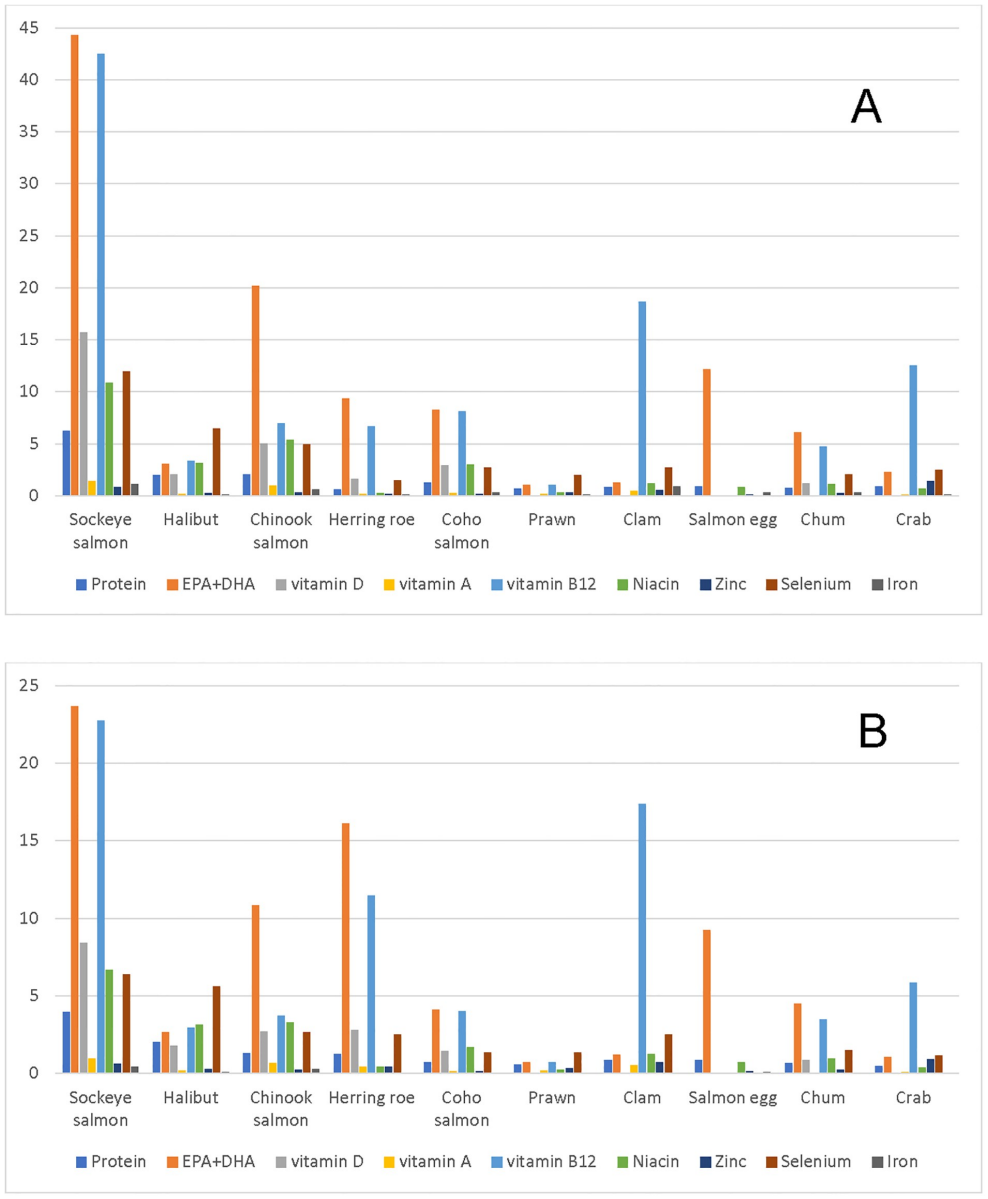
Percentage contribution of the top 10 most consumed seafood species to nutrient requirements (DRIs) in (A) men and (B) women. DRI—dietary reference intakes using recommended dietary allowance (RDA) and recommended intake (RI) for EPA+DHA.

The contributions of the top 10 seafood species to nutrient recommendations for men and women are presented in Fig 2A and 2B. Sockeye salmon provided a major contribution to the DRAs and RI. For men and women respectively, sockeye salmon alone provided 44% and 24% of EPA+DHA, 43% and 23% of vitamin B12, 16% and 8.5% of vitamin D, 11% and 7% of niacin, and 12% and 6.5% of selenium. Sockeye salmon contributed less than 10% to the protein, vitamin A, zinc and iron recommendations. Other salmon sub-species (chinook, coho, chum) and fish eggs (salmon eggs and herring roe) contributed large amounts of EPA+DHA, vitamins B12, D, niacin, and selenium in accordance to their contributions by weight. Halibut was the second-most consumed species yet contributed little to attaining requirements of selected nutrients (0.5% to 6.5%). Shellfish such as clams and crabs notably contributed to vitamin B12 requirements (19% and 13% in men, and 17% and 6% in women, respectively). Prawns yielded low intake of nutrients, contributing 0.2% to 2% to the recommended nutrient intake (Fig 2A and 2B). Intakes of protein, vitamin A, zinc, and iron from the top 10 types of seafood individually, were low among both men and women. However, the top 10 seafood collectively provided 16% and 13% of protein, 4% and 3% of vitamin A, 4.5% and 4.0% of zinc, and 3.9% and 1.9% of iron among men and women, respectively.

**Fig 3:**
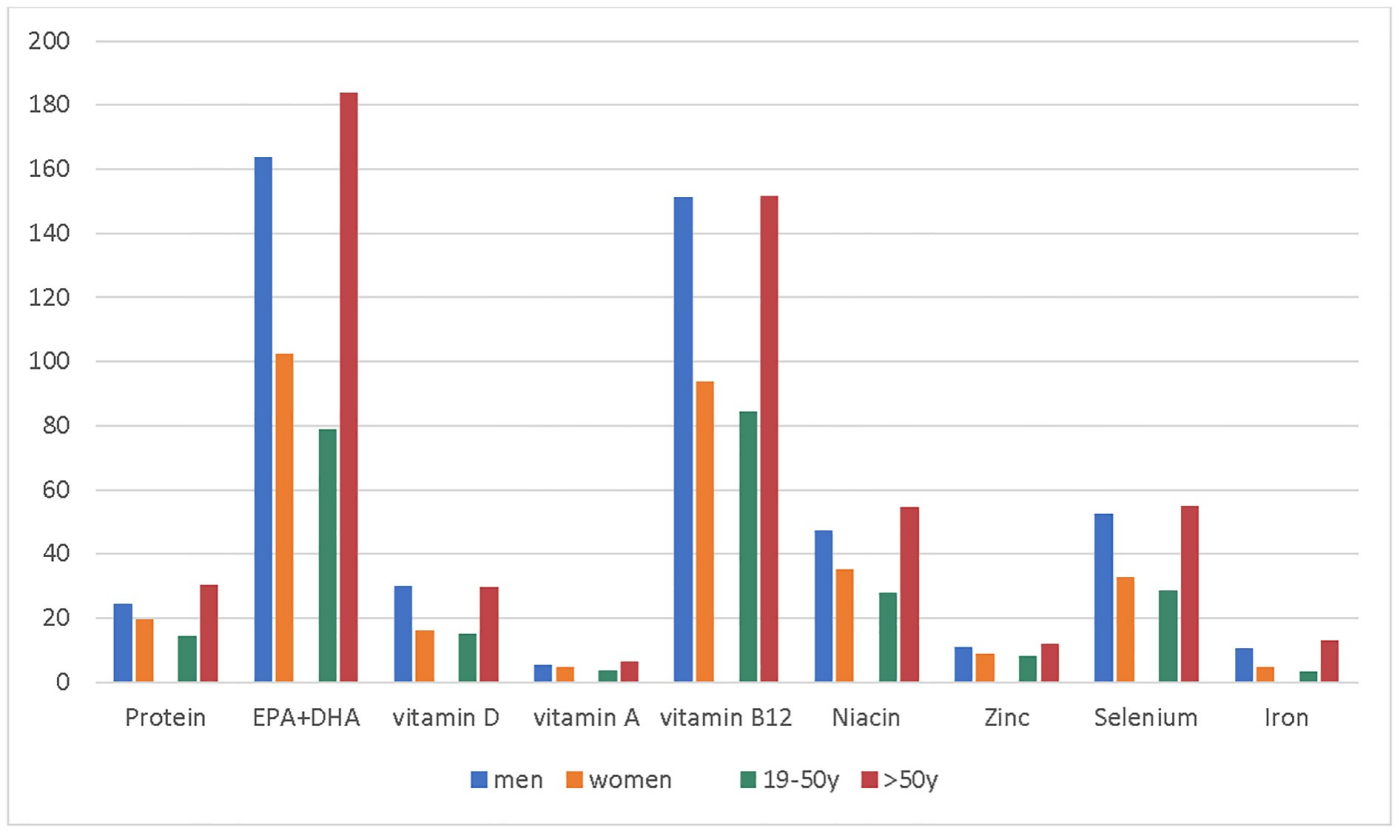
Percentage contribution of total seafood intake to nutrient requirements (DRIs) by gender and age groups. DRI—dietary reference intakes using recommended dietary allowance (RDA) and recommended intake (RI) for EPA+DHA.

The contribution of total seafood consumption to the nutrient recommendations varied by gender and age groups (Fig 3), reflecting differences in seafood consumption (by weight). Among older participants (>50 years of age), total seafood consumption provided approximately two times more nutrients compared to the younger age group (19 to 50 years of age). Likewise, men showed 1.5 to 2 times higher intakes of selected nutrients from seafood than women (Fig 3). Overall, the total seafood supplied substantial levels of nutrients contributing 79–184% to EPA+DHA, 84–152% to vitamin B12, 28–55% to niacin, and 29–55% to selenium recommendations in different gender and age groups. Seafood was found to be an excellent source of vitamin D and protein, providing between 15% and 30%, and 14% and 30%, respectively, of the age/gender-specific recommendations. The contribution of total seafood to vitamin A, zinc, and iron recommendations was low ranging from 4 to 6%, 8 to 12%, and 3% to 13%, respectively, in different age/gender groups (Fig 3).

**Fig 4:**
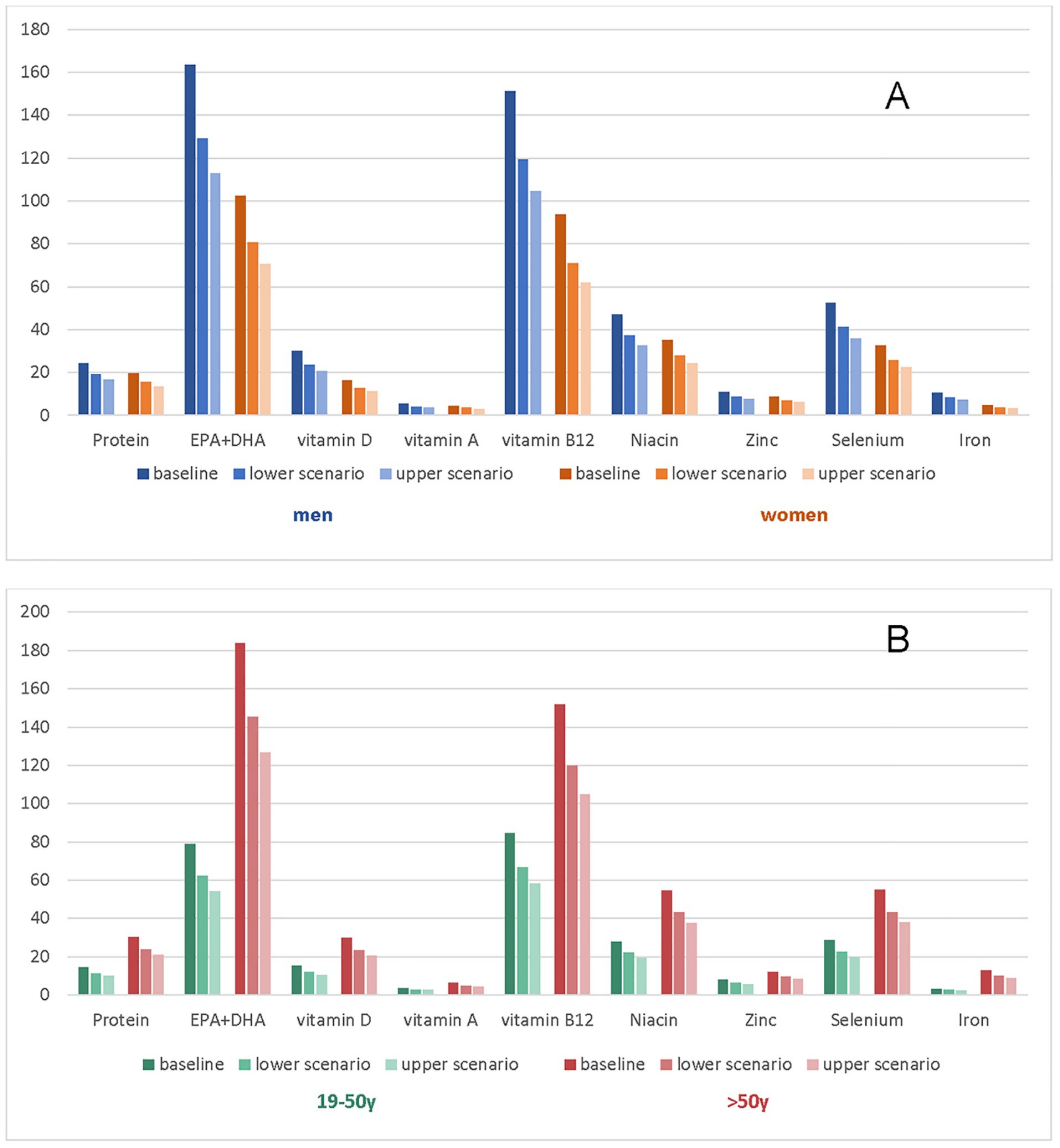
Baseline and projected percentage contributions to the DRI from total seafood in First Nations by (A) gender and (B) by age groups, under ‘strong mitigation’ (RCP 2.6) and ‘business-as-usual’ (RCP 8.5) climate change scenarios. DRI—dietary reference intakes using recommended dietary allowance (RDA) and recommended intake (RI) for EPA+DHA.

The impacts of projected climate change on seafood contributions to the DRIs in coastal FNs by age and gender groups are presented in Fig 4A and 4B. The weighted average of total seafood decline was estimated to be 21% under lower and 31% under upper climate change scenarios. Consequently, the overall contribution of seafood to the DRIs was modelled to decrease by 21 and 31% by 2050, which would significantly reduce the intake of selected nutrients. The greatest impacts on nutritional adequacy will be observed among individuals that substantially rely on traditional seafood consumption (e.g. men and older age groups), especially for those nutrients that are primarily obtained from marine sources, such as EPA+DHA, vitamin D, selenium and vitamin B12.

We projected changes in nutrient intakes after gram-to-gram substitution of the projected decline in seafood consumption with proposed alternative foods (Supporting information, S2 Table). Three hypothetical scenarios were modelled: seafood lost replaced by 1) chicken, 2) canned tuna, and 3) bread. If the chicken were used to replace 21% and 31% of reduced seafood consumption, there were no impacts on protein, niacin, and iron, while only 30% of zinc and selenium, and 50% of vitamin A intake would be replaced. However, the intake of EPA+DHA, vitamin B12, and vitamin D would be decreased by almost 100%. The substitution of traditional seafood with canned tuna would replace the amounts of protein, niacin, selenium, and iron by 100%, and half of the amount of zinc and vitamin B12; nevertheless, EPA+DHA, vitamin A intakes would be substantially reduced compared to that provided by traditional sources of seafood. Finally, if bread was used to replace traditional seafood (gram-to-gram), only iron intake would not be compromised; niacin, zinc, and selenium would be partly covered (commercially enriched) whereas protein, EPA+DHA, vitamins A, B12, and D would not.

## Discussion

The High-Level Panel of Experts (HLPE) on Food Security and Nutrition of the Committee on World Food Security, has the need to develop scenarios to understand the possible impacts of climate change on the food security and nutrition of the most vulnerable zones [2]. Canada’s Indigenous populations, particularly those residing in rural, remote, and coastal regions, disproportionally experience the effects of climate change [51,76]. Moreover, given their lower socioeconomic status, sociopolitical marginalization, and reduced access to healthy foods, they are particularly vulnerable to the consequences of climate change on food and nutrition security [77]. By modelling the effect of climate-related declines in seafood abundance on the nutritional adequacy of diets for coastal BC FNs, our results suggest that intakes of essential nutrients will decline by 21% to 31% under lower and upper scenarios of climate change by the year 2050 relative to 2000. Moreover, substitutions of seafood with selected alternative foods, such as chicken, canned tuna and bread, do not provide adequate amounts of selected nutrients.

The modelled scenarios of the impacts of the seafood production decline on nutrient intakes did not account for changes in FN population size over time. According to the most recent census, the number of FNs with registered or treaty Indian status rose by 30.8% from 2006–2016 [13]. Recent population projections revealed that between 2001 and 2011, the registered Indian population rose at an average annual rate of 2.4%, and was projected to range between 1.4 to 1.8% from 2011 to 2036 (over 25 years) [78]. For comparison, the growth rate of the non-FN population in Canada averaged 1.0% annually [78].

Using the FN population growth rate projection, we estimated the possible increase in numbers of BC FNs by 2050 relative to 2009 (BC FNFNES data collection) as well as the corresponding amounts of seafood needed to supply the growing population. With an average annual growth rate of 2.4% from 2009 to 2011 and of 1.6% from 2011 to 2050 [78], our sample of 356 individuals will increase to 693 by 2050 (almost double). In 2009, coastal BC FNs consumed about 60g/d per person of seafood; therefore, 21360 g/d of seafood (i.e., 60g/d x 356 = 21360) were needed on a daily basis. By 2050, the same amount of seafood will provide only 30.8 g/d per person (i.e., 21360 g/d/693 = 30.8 g/d) which is about half of the baseline consumption. Thus, two times more seafood will be required to supply coastal FN population in BC by 2050 relative to 2009. In contrast, our projected seafood harvest potential will decline by 21%–31% by 2050 relative to 2000, resulting in a seafood consumption rate of 21.3g/d to 24.3g/d compared to the baseline of 60g/d). The projected potential decline in seafood harvest combined with the increased demand for seafood because of population growth can result in a decrease in nutrient intake of 60%–65%. It is important to note that there has been already a steep decline in both the variety and amount of traditional food in the dietary pattern described here [79–81] which can serve to explain the limited contribution of current seafood intake for protein, vitamin A, zinc and iron. Carbon isotope measurements suggest that pre-contact, adult coastal people obtained 90% of their protein from marine sources [82]. Shellfish (a rich source of iron), and other beach foods had a much larger relative contribution [80,83] than today where few beaches remain open to harvesting because of development and contamination (sanitary or biotoxin) and foreshore leases. Other keystone species, like eulachon and eulachon grease (important contributors of vitamin A) are rarely available. For salmon alone, estimates suggest that at pre-contact, annual per capita use, at least among some Coast Salish communities was around 316 kg/year (0.9 kg/capita/day) [82,84]. This serves to highlight, while it is difficult to know with certainty what portion was used for purposes other than food, that current seafood use and its relative contributions of nutrients is at a critically low level. Nevertheless, locally-harvested seafood still supplies daily recommendations of EPA+DHA and vitamin B12, as well as substantial levels of other nutrients. Our predictions show that climate change will likely have a further impact on the seafood contribution to nutrients such as protein, EPA+DHA, vitamin D, selenium and vitamin B12. Given low or insufficient intake of selected nutrients in the total diet of BC FNs [30], the nutritional health of coastal BC FNs is highly vulnerable to this potential decline in seafood consumption related to the changing climate and species abundance. Along with the effects on diet quality, the decline in seafood consumption has detrimental impacts on mental health as well as cultural practices, language, self-determination and social cohesion.

Among a wide variety of seafood species reported to be consumed by BC FNs, salmon was the most frequently consumed, which reflects its relative abundance in BC coastal ecosystems [85] as well as its status as a cultural keystone species and preferred food for BC FNs [44]. Salmon species (collectively) supplied over 70% of the EPA+DHA recommendation, 50% of vitamin B12, 20% of vitamin D, 18% of niacin, 17% of selenium, and 10% of protein requirements. The diversity of salmon species available for harvest or consumption is an important dimension of food security and cultural stability for BC FNs. Greater population and species diversity of salmon is associated with more stable catch and longer fishing seasons [86]. In recent years, however, salmon returns have been lower than predicted, and the lowest in the past 120 years due, in part, to the warming ocean temperature [87]. With climate change affecting salmon abundance, protecting and increasing diversity among salmon populations may help provide a buffer against food insecurity among BC FNs [86]. Climate change adaptation strategies should incorporate Indigenous traditional ecological knowledge [88]

Food insecurity is an important social determinant of health for FNs and is directly related to low incomes, high costs of nutritious store-bought foods, and constraints in accessing traditional foods [40,89,90]. According to the 2008–2009 BC FNFNES, 75% of respondents observed that climate change was affecting the availability of traditional foods for harvest, while almost half the respondents reported that climate change decreased the availability of traditional foods in their households [30]. Aside from climate change, there are other barriers to traditional food consumption. Although Canadian courts have established that food, social and ceremonial fisheries of Indigenous Peoples have priority over all other uses of the resource [91], government restrictions, fleet rationalization programs, commercial and recreational harvest allocation, and other industrial activities such as forestry, hydroelectricity, mining, farming and oil/gas industries were noted by BC FNs as potential barriers to traditional food consumption [30]. Over two-thirds (68%) of FNs in BC said that all barriers, when combined, decreased access to salmon and other fish species [30].

We inevitably had to simplify the complex interactions between climate change, ecological responses, and fisheries governance impacts on fisheries and nutritional health; these assumptions can be further refined in the future to provide more detailed projections of climate change effects on FN’s food security. Firstly, the coarse resolution of the earth system model used by DBEM renders fine scale interpretation of the DBEM outputs uncertain. Thus, some of the meso-scale processes and features that may determine the fine scale distribution, changes in ecosystem productivity or abundance could not be presented in the model projection. Secondly, we only used one earth system model (GFDL ESM2M). Inter-model comparison of different earth system model outputs suggests that projected effects of climate change using GFDL ESM2M are likely to be more conservative relative to projections using other earth system models [45]. Thirdly, we assumed a linear relationship between changes in species abundance and seafood availability for FNs. However, responses of FN fishing activities, including shifts in fishing grounds, fishing effort, gear modification and changes in targeted species may also affect seafood availability under climate change. Also, changes in the exploitation status of fish stocks, fishing effort of BC FNs as well as other commercial and recreational fisheries, and the allocation of resources to different fisheries would affect the availability of fish to BC FNs directly [92]. This study assumes that these are all kept relatively unchanged in the future which is the main source of scenario uncertainty [93]. Future studies could include alternative fishing and governance scenarios to further explore these critical dimensions of access to traditional foods. Because of these limitations, our predictions cannot be viewed as a precise estimation of future fish consumption or nutrient intake. However, the broad-scale trends and comparison between climate change scenarios are robust and can be used to identify the risks to BC FNs due to climate-related changes in traditional foods and illustrate the need for the development of an adaptation plan.

Our model results show that decreases in nutrient intakes as a result of the potential decline in seafood harvest cannot be easily replaced by market foods, such as chicken, canned tuna or bread. It is important to note that the results of this modelling exercise are intended to be used by local resources managers and public health professionals as a starting point to develop adaptation plans for climate change only. There are many underlying factors such as the distribution of seafood among members in the communities, food preferences of individuals, price elasticities and access to alternative foods, etc. that will affect the relationship between fish harvest and intake. This information, as well as local data on alternative food choices, need to be collected for more realistic and relevant models. Another limitation of this exercise is the insufficient data or lack of information for sensitive subpopulations such as pregnant/lactating women and children. Since these subpopulations are the most nutritionally vulnerable, they will likely be most affected by the impacts of declining availability of seafood.

## Conclusions

Traditionally-harvested seafood remains fundamental to the contemporary diet of coastal BC FNs and provides substantial levels of essential nutrients. Dietary shifts aggravated by climate-related declines in seafood consumption may have significant nutritional and health implications for BC FNs. It is important to note that the traditional diet of coastal BC FNs has already been declining as a result of social and environmental changes and has led to the inadequate intakes of important nutrients. For example, from the Fraser River to northern BC, eulachon which was formerly a staple for coastal communities, and is known to be an excellent source of vitamin A, has almost disappeared. Similarly, bivalves, an excellent source of protein and iron, has diminished substantively. These major changes account for some of the limited changes in iron and vitamin A seen from the climate change modelling. Therefore, strategies to improve seafood harvest potential and access rights to coastal communities are needed to ensure the nutritional health and overall well-being, and to promote food security and food sovereignty in coastal FNs. In particular, more rigorous adaptations scenarios and a transformation in fisheries governance, involving participatory methodologies to incorporating Indigenous knowledge, livelihood objectives and perspectives, are needed to address the nutritional burden facing FN’s diets.

## Supporting information

S1 Table Nutrient content of top 20 most consumed seafood species (Canadian Nutrient File, Health Canada, 2015). *—μg RAE, retinol activity equivalent, “–mg NE, niacin equivalent.
(DOCX)

S2 Table Projected changes in nutrient intakes after substitution by potential alternative foods (chicken, canned tuna, and bread)^1^. ^1^—Average daily intake of nutrients, estimated base on gram-to-gram replacement by alternative foods. ^2^ –Baseline nutrient intakes from seafood based on the food frequency questionnaire of the FNFNES. ^3^ –Chicken: nutrient content is based on the most popular preparation method (chicken breast meat and skin roasted). ^4^—Canned tuna: nutrient content is based on the most reported type of canned tuna (tuna, light, canned in water, drained, unsalted). ^5^ –Bread: nutrient content is based on the most reported type of bread (bread, white, commercial, enriched). ^6^ –decline in seafood consumption under lower (21%) and upper (31%) scenarios of climate change. *—Retinol activity equivalent, RAE, "—Niacin equivalent. Bolded are the amount of nutrients not substituted by alternative foods.
(DOCX)

## Abbreviations

BC: British Columbia
BMI: body mass index
CI: confidence interval
DBEM: Dynamic bioclimate envelope model
DHA: docosahexaenoic acid
DRIs: Dietary Reference Intakes
EPA: eicosapentaenoic acid
FFQ: food frequency questionnaire
FNFNES: First Nations Food Nutrition and Environment Study
FNs: First Nations
n-3 FAs: omega-3 fatty acids
RCP: Representative Concentration Pathway
RDA: Recommended Dietary Allowance
RI: recommended intake
SD: Standard deviation
SHL: Socio-health-lifestyle questionnaire
