# Supplementary material for: Potential impacts of climate-related decline of seafood harvest on nutritional status of coastal First Nations in British Columbia, Canada
Source: PLoS One. 2019 Feb 27;14(2):e0211473. doi: 10.1371/journal.pone.0211473 (PMC6392226; doi:10.1371/journal.pone.0211473)
Supplement: S1 Table — *—μg RAE, retinol activity equivalent, “–mg NE, niacin equivalent. (DOCX) [file pone.0211473.s001.docx]

S1 Table. Nutrient content of top 20 most consumed seafood species (Canadian Nutrient File, Health Canada, 2015)

|  | Protein | EPA+DHA | vitamin D | vitamin A | vitamin B12 | Niacin | Zinc | Selenium | Iron |
| --- | --- | --- | --- | --- | --- | --- | --- | --- | --- |
| Seafood | g/100g | mg/100g | µg/100g | µg*/100g | µg/100g | mg"/100g | mg/100g | µg/100g | mg/100g |
| Sockeye salmon | 25.4 | 1230 | 13.1 | 69.0 | 5.7 | 9.7 | 0.5 | 36.5 | 0.5 |
| Halibut | 22.5 | 240 | 4.8 | 24.0 | 1.3 | 7.9 | 0.4 | 55.4 | 0.2 |
| Chinook salmon | 25.7 | 1740 | 12.9 | 149.0 | 2.9 | 14.9 | 0.6 | 46.8 | 0.9 |
| Herring roe | 22.3 | 2340 | 12.1 | 81.0 | 8.0 | 1.8 | 1.0 | 40.3 | 0.6 |
| Coho salmon | 23.5 | 1060 | 11.3 | 51.0 | 5.0 | 12.3 | 0.6 | 38.0 | 0.6 |
| Prawn | 17.4 | 176 | 0.1 | 62.0 | 0.9 | 1.7 | 1.2 | 37.8 | 0.2 |
| Clam | 25.6 | 284 | 0.1 | 171.0 | 19.5 | 8.1 | 2.7 | 64.0 | 2.8 |
| Salmon eggs | 27.0 | 2400 | 0.0 | 0.0 | 0.0 | 5.4 | 0.6 | 0.0 | 0.7 |
| Chum salmon | 21.4 | 1175 | 6.7 | 18.0 | 4.4 | 7.0 | 1.0 | 43.3 | 0.7 |
| Crab | 22.3 | 394 | 0.0 | 31.0 | 10.4 | 3.6 | 5.5 | 47.6 | 0.4 |
| Shrimp | 22.8 | 280 | 0.1 | 90.0 | 1.7 | 2.7 | 1.6 | 49.5 | 0.3 |
| Eulachon grease | 0.0 | 1000 | 0.0 | 21.0 | 0.0 | 0.0 | 0.0 | 0.0 | 0.0 |
| Pink salmon | 23.1 | 1077 | 14.5 | 20.0 | 5.0 | 11.6 | 1.0 | 39.5 | 0.8 |
| Rockfish | 22.2 | 345 | 4.6 | 5.0 | 1.6 | 7.8 | 0.4 | 76.2 | 0.4 |
| Ling cod | 22.6 | 263 | 0.0 | 17.0 | 4.2 | 6.5 | 0.6 | 46.8 | 0.4 |
| Eulachon | 14.6 | 3100 | 0.0 | 9.0 | 0.0 | 4.2 | 0.0 | 0.0 | 0.9 |
| Black cod | 17.2 | 1787 | 0.0 | 102.0 | 1.4 | 8.3 | 0.4 | 46.8 | 1.6 |
| Pacific cod | 18.7 | 160 | 0.6 | 2.0 | 2.3 | 5.2 | 0.4 | 28.0 | 0.2 |
| Basket cockle | 25.6 | 284 | 0.1 | 171.0 | 19.5 | 8.1 | 2.7 | 64.0 | 0.4 |
| Trout, any | 26.6 | 936 | 5.0 | 19.0 | 7.5 | 10.7 | 0.9 | 16.2 | 1.9 |

* - µg RAE, retinol activity equivalent, “– mg NE, niacin equivalent
