# Supplementary material for: Potential impacts of climate-related decline of seafood harvest on nutritional status of coastal First Nations in British Columbia, Canada
Source: PLoS One. 2019 Feb 27;14(2):e0211473. doi: 10.1371/journal.pone.0211473 (PMC6392226; doi:10.1371/journal.pone.0211473)
Supplement: S2 Table — 1—Average daily intake of nutrients, estimated base on gram-to-gram replacement by alternative foods. 2 –Baseline nutrient intakes from seafood based on the food frequency questionnaire of the FNFNES. 3 –Chicken: nutrient content is based on the most popular preparation method (chicken breast meat and skin roasted). 4—Canned tuna: nutrient content is based on the most reported type of canned tuna (tuna, light, canned in water, drained, unsalted). 5 –Bread: nutrient content is based on the most reported type of bread (bread, white, commercial, enriched). 6 –decline in seafood consumption under lower (21%) and upper (31%) scenarios of climate change. *—Retinol activity equivalent, RAE, "—Niacin equivalent. Bolded are the amount of nutrients not substituted by alternative foods. (DOCX) [file pone.0211473.s002.docx]

S2 Table. Projected changes in nutrient intakes after substitution by potential alternative foods (chicken, canned tuba, and bread)^1^

|  | Protein | EPA+DHA | Vitamin D | Vitamin A | Vitamin B12 | Niacin | Zinc | Selenium | Iron |
| --- | --- | --- | --- | --- | --- | --- | --- | --- | --- |
|  | g | mg | µg | µg RAE* | µg | mg NE" | mg | µg | mg |
| baseline nutrient intake2 | 13.90 | 598.72 | 3.07 | 36.65 | 2.61 | 5.74 | 0.87 | 21.17 | 0.63 |
| lower scenario6 | 10.98 | 472.99 | 2.42 | 28.95 | 2.06 | 4.53 | 0.68 | 16.72 | 0.5 |
| upper scenario6 | 9.59 | 413.12 | 2.12 | 25.29 | 1.80 | 3.96 | 0.60 | 14.61 | 0.43 |
| replaced by chicken3 |  |  |  |  |  |  |  |  |  |
| lower scenario6 | 14.74 | **472.99** | **2.44** | 32.48 | **2.10** | 6.85 | 0.81 | 19.84 | 0.63 |
| upper scenario6 | 15.14 | **413.12** | **2.13** | 30.50 | **1.86** | 7.38 | 0.79 | 19.20 | 0.63 |
| replaced by canned tuna4 |  |  |  |  |  |  |  |  |  |
| lower scenario6 | 14.20 | **473.02** | **2.57** | **28.95** | 2.43 | 6.80 | 0.78 | 26.85 | 0.69 |
| upper scenario6 | 14.34 | **413.17** | **2.34** | **25.29** | 2.35 | 7.31 | 0.74 | 29.56 | 0.72 |
| replaced by bread5 |  |  |  |  |  |  |  |  |  |
| lower scenario6 | **12.14** | **472.99** | **2.46** | **28.95** | **2.06** | 5.29 | 0.79 | 20.62 | 0.96 |
| upper scenario6 | **11.29** | **413.12** | **2.17** | **25.29** | **1.80** | 5.08 | 0.76 | 20.35 | 1.11 |

*^1^ - Average daily intake of nutrients, estimated base on gram-to-gram replacement by alternative foods*

*^2^ – Baseline nutrient intakes from seafood based on the food frequency questionnaire of the FNFNES*

*^3^ – Chicken: nutrient content is based on most popular preparation method (chicken breast meat and skin roasted)*

*^4^ - Canned tuna: nutrient content is based on most reported type of canned tuna (tuna, light, canned in water, drained, unsalted)*

*^5^ – Bread: nutrient content is based on most reported type of bread (bread, white, commercial)*

*^6^ – decline in seafood consumption under lower (21%) and upper (31%) scenarios of climate change*

** - Retinol activity equivalent, RAE, " - Niacin equivalent*

*Bolded are amount of nutrients not substituted by alternative foods*
